# Supplementary material for: A Global Screen for Assembly State Changes of the Mitotic Proteome by SEC-SWATH-MS
Source: Cell Syst. 2020 Feb 26;10(2):133–155.e6. doi: 10.1016/j.cels.2020.01.001 (PMC7042714; doi:10.1016/j.cels.2020.01.001)

Q9Y2A7 | NCKP1_HUMAN | NCKAP1 HEM2 KIAA0587 NAP1
Monomer MW [kDa]: 128.79 Monomer expected elution fraction: 38

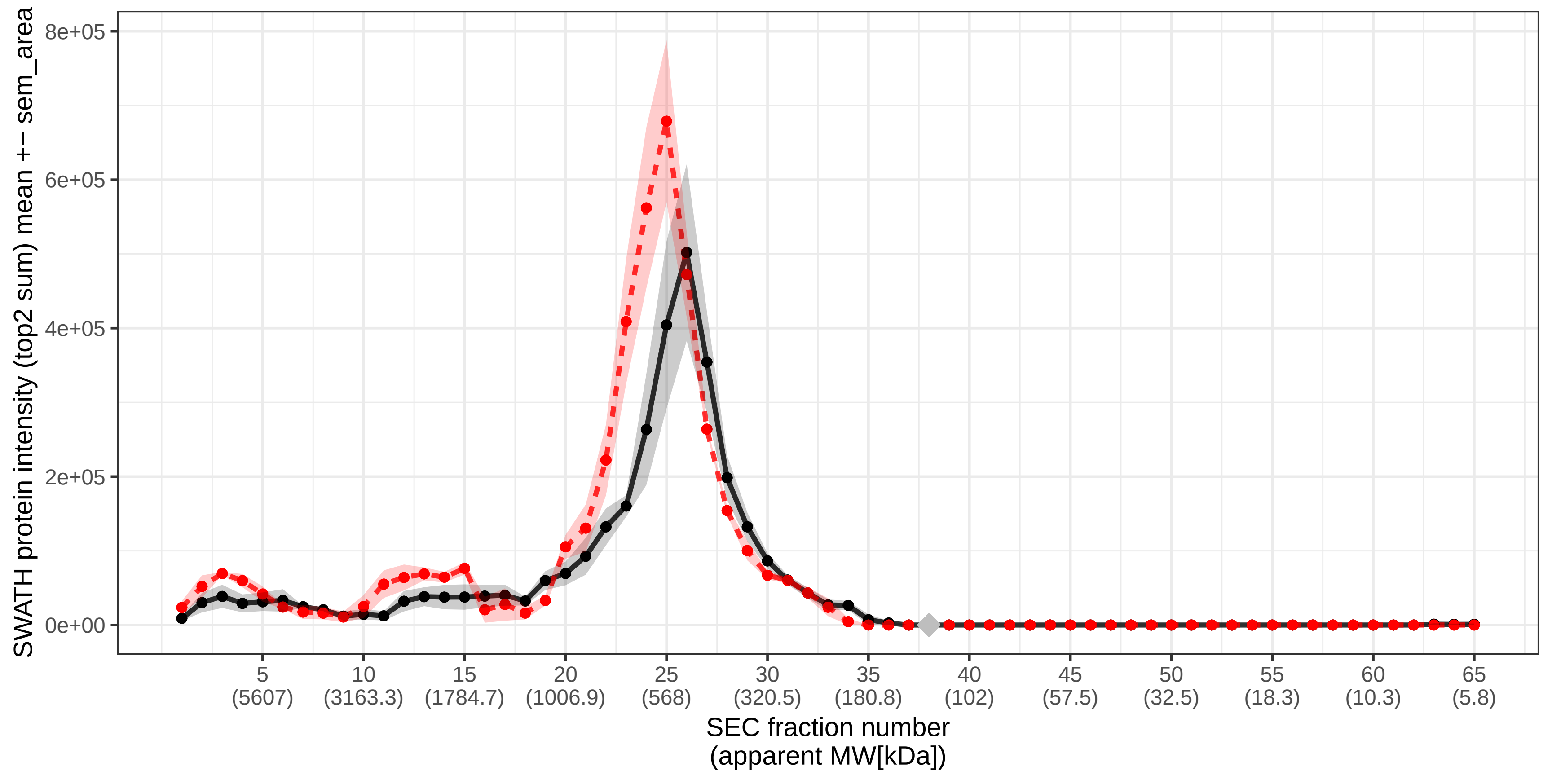

Supplement: Data S1. SEC-SWATH-MS Protein Chromatograms, Related to Figure 1 [file mmc6.zip › SECchrom_Q9Y2A7_NCKP1_HUMAN_NCKAP1_HEM2_KIAA0587_NAP1.pdf]
